# DEHP-Induced Glioblastoma in Zebrafish Is Associated with Circadian Dysregulation of PER3

**DOI:** 10.3390/toxics12120835

**Published:** 2024-11-21

**Authors:** Shuhui Men, Jiayun Xu, Zhanhong Yang, Zhenguang Yan

**Affiliations:** 1 State Key Laboratory of Environmental Criteria and Risk Assessment, Chinese Research Academy of Environmental Sciences, Beijing 100012, China; sh_men@163.com; 2 Ningbo Clinical Pathology Diagnosis Center, Ningbo 315021, China; xujiayun19@mails.ucas.edu.cn; 3 Environmental Standards Institute of Ministry of Ecology and Environment of the People’s Republic of China, Beijing 100012, China

**Keywords:** DEHP, carcinogenic effect, circadian rhythm, Period circadian regulator 3

## Abstract

DEHP is a plasticizer that is widely found in our water environment and poses a significant risk to the environment and human health. Long-term exposure to DEHP can cause endocrine disruption and interfere with the organism’s normal functioning. In order to explore the potential effects of DEHP on the development of biological brain tissues, this study used bioinformatics analysis to confirm the diagnostic and prognostic value of PER3 in gliomas and further validated the neurotoxicity of DEHP using methods such as behavioral experiments and molecular biology in zebrafish. The experimental findings revealed that the expression level of PER3 in diseased tissues was significantly lower than that in the control group. In addition, the expression level of PER3 was significantly correlated with immune cell infiltration, immune checkpoint genes, and oncogenes. Moreover, the ROC curve analysis showed that PER3 could accurately differentiate between GBM tissues and adjacent normal tissues. To further validate the neurotoxicity of DEHP, we analyzed the effects of DEHP exposure on zebrafish development and PER3 expression by behavioral experiments and molecular biology. The results showed that exposure to DEHP substantially altered both the behavioral responses and the gene expression profiles within the brain tissues of zebrafish. PCR results indicate that the expression of circadian rhythm factor PER3 was significantly reduced in the brains of zebrafish in the exposed group, and circadian dysregulation had a certain promoting effect on the development of glioma. The aim of this work was to investigate the potential effects of DEHP contamination in a water environment on organism brain development. It was demonstrated that PER3 is an effective early diagnostic marker, which is of great significance in the diagnosis and clinical prognosis of glioma, and that DEHP exposure can lead to a significant reduction in PER3 expression in zebrafish brain tissue. This study further proved that DEHP has a potential carcinogenic effect, which adds scientific evidence to the carcinogenicity study of DEHP.

## 1. Introduction

Di-2-ethylhexyl phthalate (DEHP) is a plasticizer that is commonly used in the production of food packaging and medical supplies. Due to its unstable covalent bond, it can easily be released into the environment from plastic products, resulting in environmental estrogenic pollution. The direct sources of DEHP in the water environment are the discharge of industrial and domestic wastewater; the indirect sources are DEHP in the atmosphere, which enters the water body through wet deposition during long-range transport, and DEHP in the soil, which enters the water body through surface runoff and other pathways [1,2]. Studies have shown that long-term exposure to plasticizers can affect lipid metabolism in living organisms and cause hormone secretion disorders and neurodevelopmental abnormalities [3,4]. Neuroinflammation caused by overactivation of neuroimmune cells due to PAEs has been extensively studied in the field of toxicology [5]. Zhou et al. [6] discovered that DBP can cause learning disabilities and anxiety behaviors in mice by activating microglia in hippocampal neurons. Similarly, Win Shwe et al. [7] exposed mice to DEHP and found that it may induce neuroinflammation by modulating hypothalamic neuroimmune biomarkers. In addition, some studies have proposed a possible link between DEHP exposure and cancer development, particularly by affecting telomere length as one of the possible mechanisms [8]. Moreover, some studies have observed that chronic exposure to DEHP induces carcinogenic effects in rodents [9].

Yi Qing et al. [10] found that prenatal PAE exposure may affect circadian regulation in adult rats by altering the expression of circadian-related genes in the SCN nuclear region of the hypothalamus. Circadian rhythms are crucial for maintaining the health of biological organisms as they regulate a wide range of physiological functions, including metabolism, sleep, endocrine, immune, and cardiovascular functions [11,12]. In addition, there is a correlation between circadian rhythms and tumor development. Studies have shown that the dysregulation of CRY1 in mice has been linked to the development of gliomas, indicating that circadian dysregulation contributes to brain tissue development [13,14]. It has been shown that circadian dysregulation can accelerate tumor development through endocrine mechanisms such as altered clock gene expression and suppression of melatonin secretion [15]. Furthermore, circadian rhythms may indirectly affect psychological recovery in cancer patients through clusters of cancer-related symptoms [16].

Additionally, the Period gene family of genes encodes circadian components related to motor activity and metabolism. Specifically, dysregulation of Period circadian regulator 3 (PER3) has been associated with the development of a variety of malignancies, including prostate, colorectal, and hematopoietic malignancies [17]. It was also shown that breast cancer susceptibility was significantly increased in PER3-deficient mouse models [18]. While little research has been conducted on the role of them in brain tissue development. Currently, the International Agency for Research on Cancer (IARC) of the World Health Organization has classified DEHP as a group 2B carcinogen, and there is limited evidence for the carcinogenicity of DEHP in humans and insufficient evidence for its carcinogenicity in experimental animals. Consequently, the aim of this study is to investigate the diagnostic and prognostic role of PER3 in glioblastoma multiforme (GBM) and to provide a new scientific basis for the carcinogenicity of DEHP.

## 2. Materials and Methods

### 2.1. Bioinformatics Data Collection

The RNAseq data and corresponding clinicopathologic information of 706 GBM patients were obtained from the Glioma Project of the TCGA database (https://portal.gdc.cancer.gov/; accessed on 23 November 2023) in the FPKM (fragments per kilobase per million) format. Subsequently, the RNA sequencing data were converted to the TPM (transcripts per kilobase million) format. Furthermore, three additional GBM datasets were acquired from the GEO database (https://www.ncbi.nlm.nih.gov/geo/; accessed on 23 November 2023).

### 2.2. Bioinformatics Analysis of Period3 mRNA Expression Levels

The mRNA expression levels of PER3 were compared and analyzed in 33 human cancers from the TCGA database as well as in normal tissue samples. Additionally, the expression of PER3 in glioblastoma samples was analyzed using gene expression data from the GSE29458, GSE22866, and GSE14805 datasets from the GEO database. The case samples were divided into high- and low-expression groups to analyze the differentially expressed genes (DEGs) between the two groups based on the median expression value of PER3. A threshold parameter of absolute value of logarithmic fold change (Log2FC) greater than 1.5 and *p*-value less than 0.05 was used. Volcano and heat maps were visualized using the “ggplot2” (v3.3.6) R package.

### 2.3. Functional Enrichment Analysis

Gene symbols were converted to Entrez IDs using the R package “org.hs.egg.db” (v3.10.0). Functional annotation and gene set enrichment analysis (GSEA) of differential genes were performed using the R package “ClusterProfiler” (v3.14.3). The reference gene sets used in the GSEA analysis were organized from the MSigDB database (https://www.gsea-msigdb.org/gsea/msigdb/index.jsp; accessed on 29 November 2023), and c2.cp.v7.2.symbols.gmt was selected. We screened 1518 gene sets that were significantly enriched based on the threshold parameters of FDR less than 0.25 and *p* adjust less than 0.05.

### 2.4. Correlation Analysis of PER3 Expression Level, Immune Cell Infiltration and Immune Cell Markers

The immune cell tumor infiltration status was assessed using the ssGSEA algorithm from the “GSVA” (v1.34.0) R package, which includes 24 types of immune cells, such as neutrophils, macrophages, Th2 cells, NK CD56dim cells, TFH, Tem, Tgd, and Tcm. Spearman correlation analysis was performed to determine the relationship between PER3 expression levels, immune cell infiltration status, and immune cell markers. Furthermore, we analyzed the correlation between PER3 expression levels and immune checkpoint genes as well as oncogenes in GBM samples from the TCGA database using the R package “ggplot2” (v3.3.6). A correlation was considered significant if the *p*-value was less than 0.05.

### 2.5. Analysis of DNA Methylation Status and Genetic Alterations

The MethSurv database (https://biit.cs.ut.ee/methsurv/; accessed on 11 January 2024) was used to analyze the DNA methylation status of the CpG site of the PER3 gene in the GBM-TCGALGG dataset. The prognostic value of PER3’s CPG methylation status was also assessed in GBM samples, as well as its relationship with overall survival (OS) in GBM.

The PER3 genome changes were analyzed using cBioPortal (https://www.cbioportal.org/; accessed on 11 January 2024) [19,20] in three datasets: Mayo Clinic, Clinical Cancer Research 2020; CPTAC, Cell 2021; and TCGA, Firehose Legacy. The prognostic significance of PER3 gene alterations was determined by K-M survival curve analysis and log-rank test. A *p*-value of less than 0.05 was used as the criterion for a significant difference.

### 2.6. Correlation Analysis of PER3 Expression Level and Pathologic Characteristics

The TCGA-GBMLGG dataset was used to obtain pathological data of GBM patients, which included overall survival (OS), disease-specific survival (DSS), and progression-free interval (PFI). The statistical data were compared between the high and low expression groups in various pathological parameters such as age, WHO classification, histological type, OS events, DSS events, PFI events, 1p/19q deletion or not, IDH type, and tumor efficacy assessment using the “ggplot2” (v3.3.6) R package. The Shapiro–Wilk normality test was used to analyze normally distributed data (*p* < 0.05). The study utilized logistic regression analysis to assess the correlation between PER3 expression levels and the pathological characteristics of patients with GBM.

### 2.7. Prognostic Implications of PER3 Expression in Brain

In this study, patient survival data from the TCGA-GBMLGG were analyzed to determine the survival outcomes of GBM patients based on the expression levels of PER3. The analysis was performed using K-M survival curve analysis and univariate and multivariate Cox regression analysis. Diagnostic ROC curves and time-dependent ROC curves were plotted using the “pROC” (v1.18.0.), “timeROC” (v0.4), and “ggplot2” (v3.3.6) R packages. Nomogram modeling analysis was also performed to evaluate the predictive value of PER3 expression levels for GBM. Kaplan–Meier survival curves were utilized to conduct prognostic analysis on subgroups of patients with glioblastoma (GBM). The results included sample size, confidence intervals, risk ratios, and *p*-values.

### 2.8. Chemicals and Test Solutions

Di-2-ethylhexyl phthalate (CAS: 117-81-7, purity 99.5%; Product No. D201154) was purchased from Sigma-Aldrich (Darmstadt, Germany). The stock DEHP solutions were prepared with acetone (CAS: 67-64-1, purity 99.5%; Product No. 10000418; Sinopharm Chemical Reagent Co., Ltd. Shanghai, China). The acetone content in the final exposure solution is less than 5%.

### 2.9. Breeding of Zebrafish and Collection of Embryos

The AB zebrafish (*Danio rerio*) were purchased from the China Zebrafish Resource Center (Wuhai, China) and reared under the following conditions: temperature of 28 ± 0.5 °C, light/dark cycle of 14 h/10 h, and feeding of fresh plumpy shrimp twice a day.

The night before fertilized eggs were collected, males and females were placed in a spawning box in a 2:1 ratio with a baffle in the middle; the baffle was removed immediately after light exposure the following day, and eggs were collected after 30 min of spawning. Embryos/larvae were raised in E3 medium [21] (294.0 mg/L CaCl_2_·2H_2_O, 123.3 mg/L MgSO_4_·7H_2_O, 64.7 mg/L NaHCO_3_, 5.7 mg/L KCl) in a Petri dish and placed in an incubator at 28 °C.

All zebrafish husbandry and experimental procedures of our study were performed in accordance with the guidelines of the Animal Care and Use Committee of Chinese Research Academy of Environmental Sciences.

### 2.10. Exposure Experimental Protocol

Zebrafish were divided into control group, low concentration group (50 μg/L), medium concentration group (500 μg/L), and high concentration group (1500 μg/L), where 50 μg/L was a reference to the health baseline value of DEHP [22]. A 500 μg/L amount refers to the environmental background value of the contaminated areas in China [2,23,24], and in order to better observe the toxicological mechanism, a high concentration value of 1500 μg/L is set. Each group was set up with 3 parallel groups, and 20 zebrafish in each parallel group were exposed to DEHP solution for 30 days. A new exposure solution was replaced every 24 h during the experiment.

At the end of the exposure, zebrafish embryos were obtained according to the methods described in Section 2.2. The morphological observations and behavioral experiments were performed at 5 dpf and 7dpf to observe the effects of DEHP exposure on zebrafish offspring, respectively.

### 2.11. Behavioral Experiments on Zebrafish Larvae

To investigate the effects of DEHP exposure on the behavioral patterns of zebrafish offspring, we controlled the light intensity of the behavioral experimental apparatus and observed the responses of zebrafish larvae in a dark environment and under alternating light and dark stimuli. Ten larvae per group were selected for behavioral observations. First, 7 dpf zebrafish larvae were placed in a well plate and acclimatized to a dark environment for 2 min. Then, their movement trajectory and residence time in the dark environment were recorded for 10 min. Next, the zebrafish were acclimatized to a bright environment for 2 min, followed by alternating light and dark stimuli performed at a frequency of 5 min for a cycle of 3 times. The study counted the average movement speed of larvae during a 30 s interval and observed behavioral differences between exposed and control zebrafish.

### 2.12. Quantitative Real-Time PCR

Zebrafish were euthanized using MS-222 anesthetics, followed by dissection to obtain intact zebrafish brain tissue. Each group contained 3 replicates, and each sample contained 5 brain tissues. Total RNA was extracted from each sample using MolPure^®^ Cell/Tissue Total RNA Kit (YEASEN, 19221ES50), and the RNA samples were reverse transcribed using Hifair^®^ III qPCR First-Strand CDNA Synthesis Ready-to-Use Pre-Mix (YEASEN, 11141ES60) (with gDNA removal) and cDNA solution was obtained. A 20 μL system for qPCR was prepared using the SYBRGreen MasterMix (YEASEN, 11184ES08) fluorescent dye assay. The reaction was carried out using a two-step PCR program, which included denaturation at 95 °C for 10 s, annealing/extension at 60 °C for 31 s, and cycling for 40 times. Prior to this, pre-denaturation at 95 °C for 2 min was performed to promote the template to unravel the secondary structure and revitalize the hot starter enzyme. Primers were designed with Primer3plus software (3.3.0) and analyzed for specificity using NCBI Blast online.

## 3. Results

### 3.1. Expression of PER3 in Multiple Cancers Including GBM

PER3 expression was analyzed in 33 cancer datasets from the TCGA database. In 23 cancers, PER3 showed significant downregulation. Furthermore, PER3 expression was significantly reduced in glioblastoma tissues from the GSE29458, GSE22866, and GSE14805 datasets compared to the corresponding controls.

**Figure 1:**
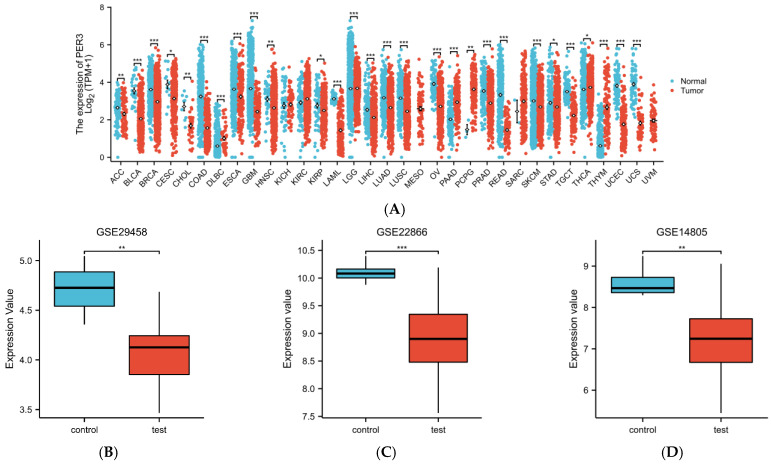

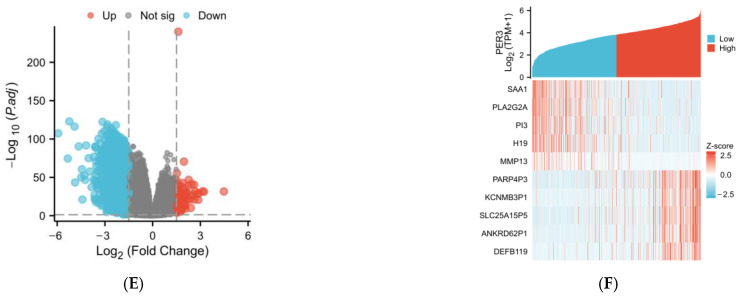
PER3 expression is significantly downregulated in several cancers, including GBM. (**A**) TCGA database analysis showed the expression levels of PER3 in 33 cancer tissues and their corresponding surrounding normal tissues. * *p* < 0.05; ** *p* < 0.01; *** *p* < 0.001. (**B**–**D**) The expression levels of PER3 in cases (**B**) GSE29458, (**C**) GSE22866, and (**D**) GSE14805 in GBM tissues had significantly higher expression levels than normal brain tissues. (**E**,**F**) The 706 GBM patients in the TCGA-GBMLGG database were categorized into high-expression and low-expression groups based on the median PER3 expression level. (**E**) Volcano plot and (**F**) heat map demonstrating the expression levels of specific mRNAs in GBM patients.

The 706 GBM patients were categorized into high- and low-expression groups based on the median PER3 expression value. The threshold parameters used were an absolute value of log fold change greater than 1.5 and a *p*-value of less than 0.05. A total of 2284 genes were found to be significantly different compared to the PER3 expression value, with 2125 genes being significantly downregulated and 158 genes being significantly upregulated. The single gene co-expression heatmap in Figure 1F displays ten of the most significantly differentiated genes.

### 3.2. Functional Enrichment Analysis of PER3-Related Differentially Expressed Genes in GBM

**Figure 2:**
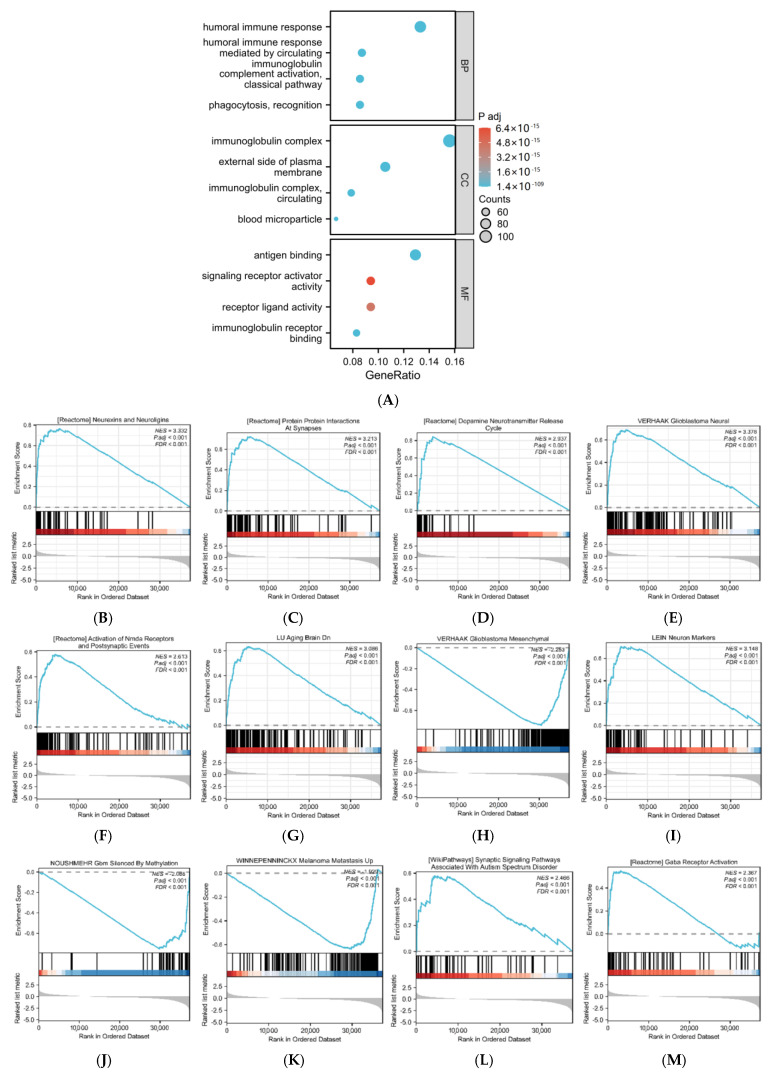
Functional enrichment analysis of differentially expressed genes in GBM based on PER3 expression levels. (**A**) GO functional enrichment analysis of PER3-associated differential genes showing enrichment for biological process (BP), cellular component (CC), and molecular function (MF). (**B**–**M**) GSEA genomic enrichment analysis of altered signaling pathways in GBM tissues based on PER3-related differential genes in GBM high and low expression groups.

Functional annotation of differential genes associated with PER3 in GBM patients was performed using ClusterProfiler R. GO enrichment analysis results revealed highly enriched biological processes, cellular components, and molecular functions (*p* < 0.05), as shown in Figure 2A and Supplementary Materials. The biological processes that ranked the highest were phagocytosis, humoral immune response, complement activation, and immune response mediated by circulating immunoglobulin. The cellular component GO terms that were most enriched were immunoglobulin complex, blood microparticle, and the external side of the plasma membrane. The molecular functions that were most enriched were antigen binding, immunoglobulin receptor binding, receptor ligand activity, and signaling receptor activator activity.

The Gene Set Enrichment Analysis (GSEA) can reveal functional and pathway distinctions between the tumor and normal groups. Compared to general difference analysis (GO and KEGG), GSEA analysis does not require the delineation of a difference multiplicity threshold. Instead, it orders and assigns enrichment to genes as a whole based on their expression. This approach more comprehensively and intuitively demonstrates the overall expression trend of the gene set [25]. The study found that the set of PER3-related differential genes was significantly enriched for genes related to neural function, including neurexins and neuroligins (NES = 3.332, padj < 0.001, FDR < 0.001), protein interactions at synaptic level (NES = 3.213, padj < 0.001, FDR < 0.001), neuron markers (NES = 3.148, padj < 0.001, FDR < 0.001), dopamine neurotransmitter release cycle (NES = 2.937, padj < 0.001, FDR < 0.001), activation of Nmda receptors and postsynaptic events (NES = 2.613, padj < 0.001, FDR < 0.001), GABA receptor activation (NES = 2.367, padj < 0.001, FDR < 0.001), etc. In addition, PER3-associated DEGs were also enriched in neurological disease-associated gene sets, such as glioblastoma neural (NES = 3.378, padj < 0.001, FDR < 0.001), aging brain Dn (NES = 3.086, padj < 0.001, FDR < 0.001), glioblastoma mesenchymal (NES = −2.253, padj < 0.001, FDR < 0.001), melanoma metastasis UP (NES = −1.929, padj < 0.001, FDR < 0.001), GBM silenced by methylation (NES = −2.086, padj < 0.001, FDR < 0.001), synaptic signaling pathways associated with autism spectrum disorder (NES = 2.466, padj < 0.001, FDR < 0.001), etc.

Figure 2B–M displays the enrichment score in the upper part of the result graph. A higher ES value indicates that the genes are enriched in the pathway rather than dispersed. A positive NES indicates head enrichment, with core molecules concentrated in this gene tending to be upregulated. Conversely, a negative NES indicates tail enrichment, with core molecules concentrated in this gene tending to be downregulated. The position of each gene is marked with lines in the images, while the vertical lines represent the concentration of molecules in the gene.

### 3.3. PER3 Expression Levels Correlate with Multiple Immune Cell Infiltration in Glioblastoma Tissues

**Figure 3:**
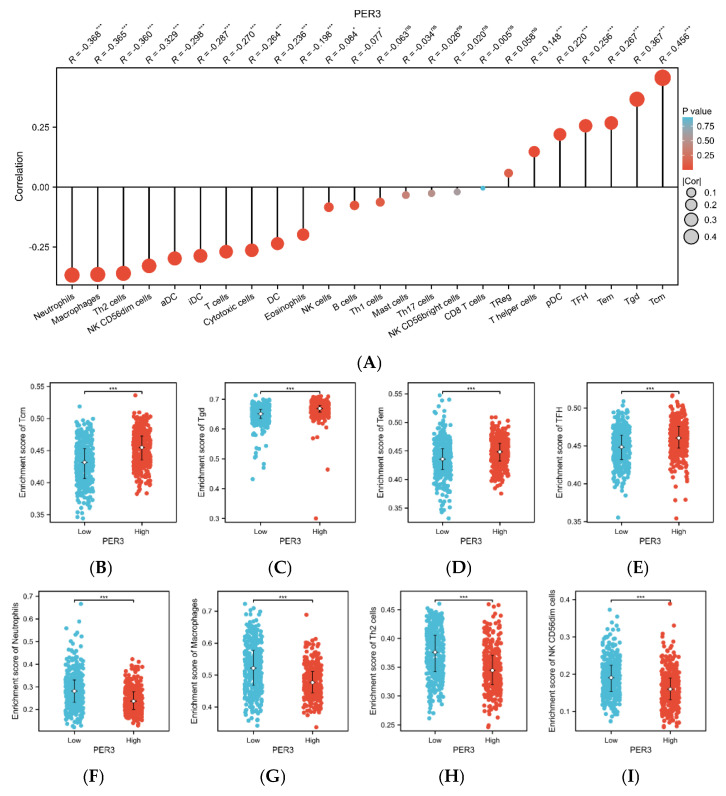
Correlation analysis of immune cell infiltration with PER3 expression in GBM. (**A**) Spearman’s correlation analysis of the infiltration level of 24 immune cells with the expression level of PER3 in GBM tissues. (**B**–**I**) Infiltration levels of (**B**) central memory cells, (**C**) γδ T cells, (**D**) effector memory T cells, (**E**) follicular helper T cells, (**F**) neutrophils, (**G**) macrophages, (**H**) type 2 helper T cells, and (**I**) CD56dim NK cells in the high and low expression groups. ns, *p* ≥ 0.05; * *p* < 0.05; *** *p* < 0.001.

The single-sample Gene Set Enrichment Analysis (ssGSEA) was applied to evaluate the infiltration of 24 immune cells in GBM tissues [26,27]. The relationship between PER3 and immune cell infiltration was assessed through Spearman correlation analysis. The figure shows that PER3 expression levels were negatively correlated with neutrophils (R = −0.368, *p* < 0.001), macrophages (R = −0.365, *p* < 0.001), Th2 cells (R = −0.360, *p* < 0.001), and NK CD56dim cells (R = −0.329, *p* < 0.001). Conversely, PER3 expression levels were negatively correlated with Tcm cells (R = 0.456, *p* < 0.001), Tgd cells (R = 0.367, *p* < 0.001), Tem cells (R = 0.267, *p* < 0.001), and TFH cells (R = 0.256, *p* < 0.001). We next evaluated the relationship between the infiltration levels of the above several immune cells in different PER3 expression groups, and the results obtained were consistent with the analysis in Figure 3A.

### 3.4. Correlation of PER3 Expression Levels with the Expression of Immune Checkpoint Genes and Oncogenes in Glioblastoma Tissue

CD276, HLA-A, and TNFRSF4 are immune checkpoint proteins that are associated with tumor immune evasion. Their receptors are co-stimulatory factors that are associated with T cell immunity. High expression of immune checkpoint molecules can suppress the body’s immune function.

**Figure 4:**
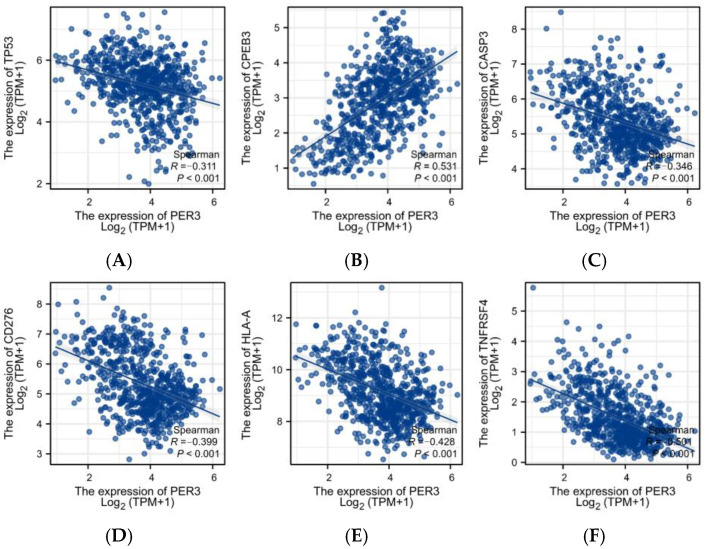
Correlation analysis of PER3 expression level with oncogene (**A**) TP53, (**B**) CPEB3, (**C**) CASP3, and immune checkpoint (**D**) CD276, (**E**) HLA-A, and (**F**) TNFRSF4 expression level in glioblastoma tissues.

Figure 4 exhibits the correlation between the expression levels of PER3 and the above genes in GBM. It was found that the expression level of the PER3 gene was positively correlated with that of CPEB3 and negatively correlated with the expression of other oncogenes and immune checkpoint proteins in GBM samples in the TCGA database. These results further demonstrate the potential diagnostic role of PER3 in GBM.

### 3.5. PER3 Gene Methylation Status Is Associated with Prognosis in Patients with Glioblastoma

**Figure 5:**
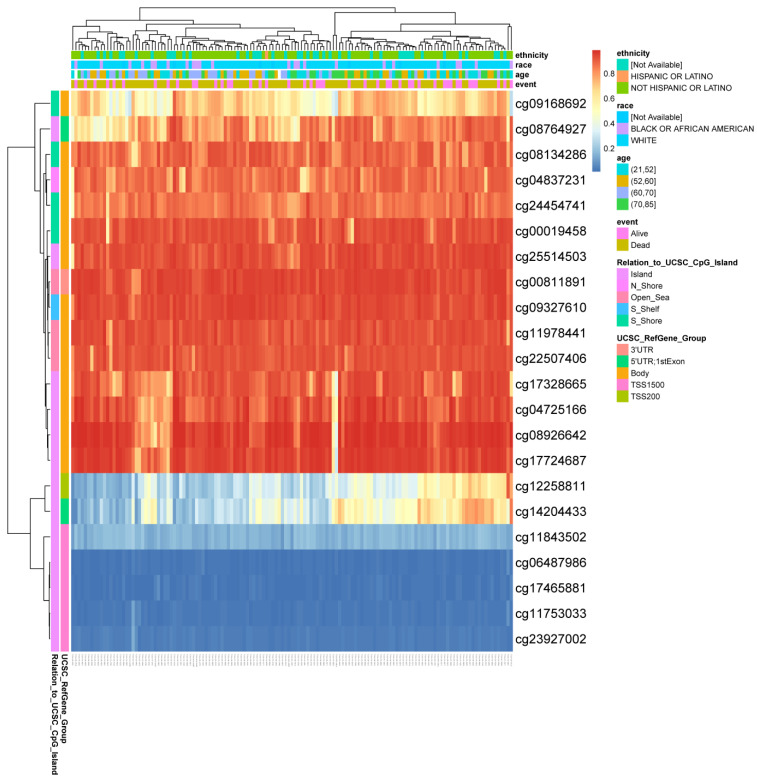
DNA methylation level of PER3 gene is associated with prognosis of GBM.

In the study, the DNA methylation level of the PER3 gene and the prognostic value of the CpG islands of the PER3 gene in GBM were analyzed using the MetSurv tool (https://biit.cs.ut.ee/methsurv/; accessed on 11 January 2024). As shown in Figure 5, the results revealed methylation alterations in 22 CpG islands, with cg12258811, cg14204433, cg11843502, cg06487986, cg17465881, cg11753033, and cg23927002 showing reduced DNA methylation levels. We then analyzed the prognostic impact of methylation levels at different CpG islands on GBM patients based on the KM curves of PER3 methylated sites (Table 1). The results showed that the methylation levels of eight CpG islands (cg06487986, cg08764927, cg11978441, cg12258811, cg14204433, cg17465881, cg17724687, cg23927002) were correlated with the prognosis of GBM patients (*p* < 0.05). Specifically, reduced methylation levels of PER3 at these CpG sites were associated with lower overall survival in GBM patients compared to those with higher PER3 methylation levels.

### 3.6. PER3 Expression Levels Correlate with Multiple Clinicopathologic Features of Brain GBM

**Figure 6:**
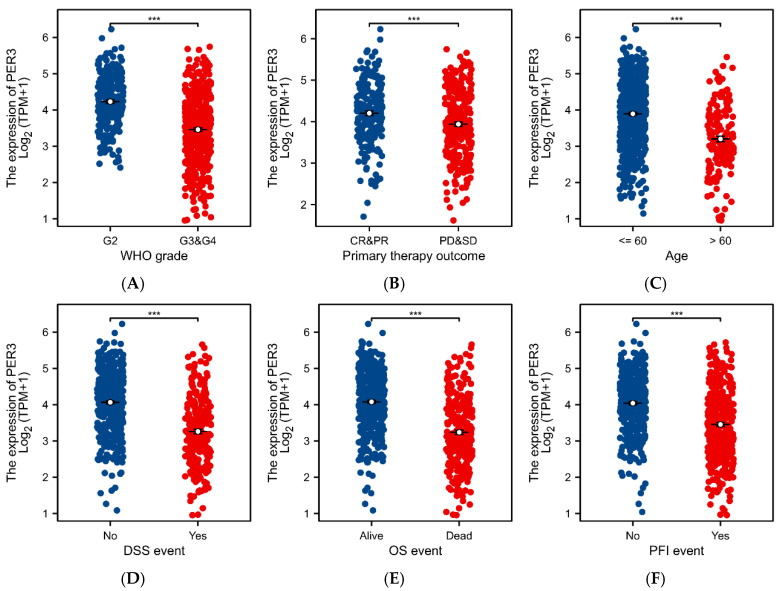

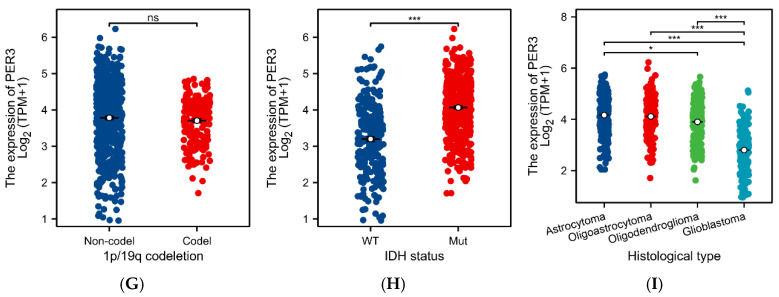
PER3 expression levels correlate with a variety of clinical case characteristics in GBM patients. (**A**–**I**) The correlation analysis between PER3 expression level and (**A**) WHO grade, (**B**) Primary therapy outcome, (**C**) Age, (**D**) DSS, (**E**) OS, (**F**) PEI, (**G**) 1p/19q codeletion, (**H**) IDH status, (**I**) Histological type. ns, *p* ≥ 0.05; * *p* < 0.05; *** *p* < 0.001.

The relationship between clinical case characteristics of GBM patients and PER3 expression levels based on the TCGA-GBMLGG dataset is shown in Table 2. Significant differences were found between GBM patients with high and low PER3 expression in terms of histopathological type, WHO grade, IDH status, primary therapy outcome, OS events, DSS events, and PFI events. No significant correlation was found in terms of patients’ ethnicity (Figure 6). In glioblastoma tissues, patients with high tumor progression exhibited lower levels of PER3. Logistic regression analysis revealed significant correlations between PER3 expression levels and WHO grade, IDH status, primary therapy outcome, and patient age (Table 3).

### 3.7. PER3 Is a Potential Prognostic and Diagnostic Biomarker for Brain GBM

**Figure 7:**
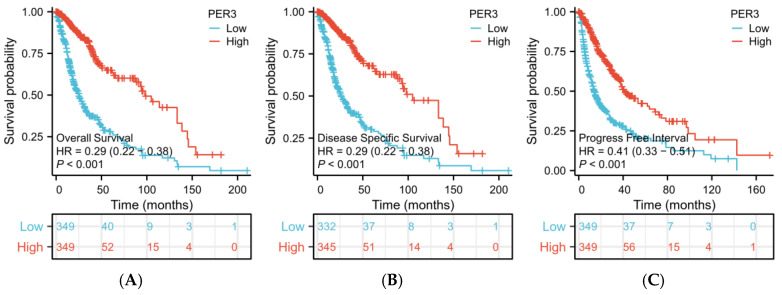
PER3 exhibits high prognostic predictive value in patients with GBM. k-M plots show the differences in (**A**) overall survival, (**B**) disease-specific survival, and (**C**) progression-free intervals in patients with GBM in the high and low PER3 expression groups. *p* < 0.05 was statistically significant.

The analysis of the K-M survival curve (Figure 7) revealed that patients with glioblastoma multiforme who had low PER3 expression levels had significantly lower overall survival, disease-specific survival, and progression-free interval compared to those with high levels of PER3 expression. The results of the multifactorial Cox regression analysis, presented in Table S2, indicate that PER3 is an independent risk factor for predicting OS (HR: 0.853, *p* < 0.001). However, it is not a risk factor for DSS and PFI. The HR value less than 1 suggests that low expression of the PER3 gene is detrimental to the overall survival of GBM patients.

Additionally, age, IDH type, and tumor efficacy assessment were independent predictors of OS, DSS, and PFI. The WHO classification was also clinically important in predicting OS and DSS.

**Figure 8:**
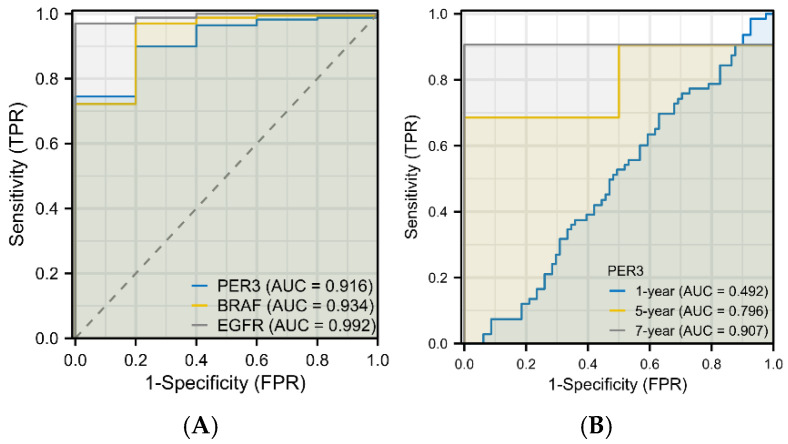
PER3 exhibits excellent diagnostic and prognostic performance in GBM (**A**) three-factor diagnostic ROC curve. (**B**) Time-dependent ROC curves predicting 1-, 5-, and 7-year survival rates in glioblastoma patients based on PER3 expression levels.

The diagnostic value of PER3 expression level was analyzed using the receiver operating characteristic (ROC) curve (Figure 8). An area under the curve (AUC) value greater than 0.5 proved that the variable was diagnostic in predicting the outcome. The diagnostic ROC plot showed that the AUC value of PER3 as a diagnostic factor was 0.916. This indicates that the PER3 expression level can accurately differentiate the tumor tissue from the surrounding tissue adjacent to the tumor. Additionally, basic fibroblast growth factor receptor (BAFR) and epidermal growth factor receptor (EGFR), which are common molecular markers for glioblastoma, were selected as diagnostic factors for graphing. The AUC values were above 0.9, indicating that the diagnostic effect of PER3 in predicting the outcome was consistent with the common prognostic biomarkers of glioblastoma.

Time-dependent ROC curve analysis demonstrated that the AUC values for predicting 5- and 7-year survival rates of GBM patients based on PER3 expression levels were both greater than 0.7.

### 3.8. DEHP Exposure Effects on PER3 Gene Expression in Zebrafish Brain

**Figure 9:**
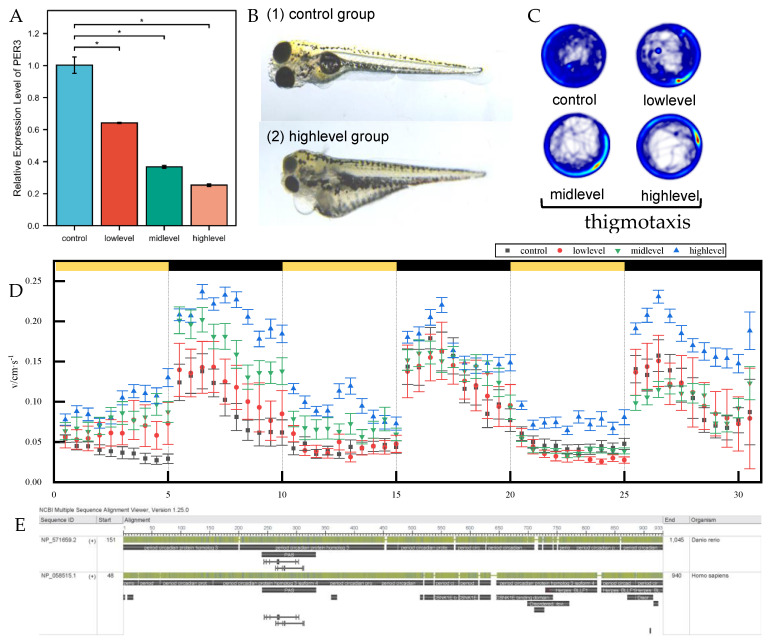
Toxic effects of DEHP on zebrafish. (**A**) Effects of DEHP exposure on the differential expression of PER3 gene in the zebrafish brain. * *p* < 0.05. (**B**) Effects of DEHP exposure on zebrafish growth and development. (**C**) Effects of zebrafish exposure on spontaneous locomotion of juvenile fish. (**D**) Behavioral effects of light and dark cyclic stimulation on different subgroups of zebrafish. (**E**) Analysis of the homology of zebrafish and human PER3 protein.

The PER gene family plays a crucial role in regulating circadian rhythms. This study examined the effects of exposure to different concentrations of DEHP solution on adult zebrafish. After 30 days of exposure, the expression of the circadian factor PER3 was significantly downregulated in the zebrafish brain (Figure 9A), confirming the regulation of circadian rhythm-related genes in zebrafish by DEHP. Protein homology analysis revealed homologous similarity between zebrafish and human PER3 proteins (Figure 9E). Additionally, zebrafish are genetically similar to humans, with a genetic conservatism of 85%. This high degree of genetic similarity makes zebrafish valuable as a replacement for humans in disease or drug testing. Thus, the differential expression of the PER3 gene in zebrafish provides valuable information for studying human diseases.

### 3.9. Effects of DEHP Exposure on the Growth, Development, and Behavioral Patterns of Larval Zebrafish

The study observed the development of zebrafish offsprings over a 5-day period and found that DEHP exposure had a significant impact on their overall morphological development. This was mainly characterized by developmental delays, curvature of the body axis, and pericardial edema (Figure 1B).

To investigate the impact of DEHP on zebrafish circadian rhythms, we analyzed the behavior of 7 dpf zebrafish larvae. We observed their spontaneous locomotor activity in the dark and their behavior under alternating light and dark conditions. We also measured their tendency to reflect their anxiety levels in unfamiliar environments (Figure 1C). Compared to the control group, the exposed group showed a significant increase in residence time in both the peripheral and central zones. This suggests that exposure to DEHP worsened anxiety levels in zebrafish larvae.

Furthermore, the behavioral variations of zebrafish in diverse lighting conditions can serve as a foundation for identifying neurological disorders. Additionally, the response of zebrafish to changes in light and darkness can demonstrate its capacity to regulate circadian rhythms [28]. The findings indicate that exposure to DEHP can impact the reactivity of zebrafish larvae when exposed to alternating light and dark stimuli (Figure 1D). With an increase in the number of cycles, the swimming speed of the control group tends to stabilize under different conditions. However, the speed of the exposed group of juvenile fish varies more and is concentration-dependent.

## 4. Discussion

PER3 is a circadian regulator that can influence cell cycle, growth, and differentiation. The study found that PER3 was overexpressed in 22 out of 33 common human cancers. Researchers [18] conducted cell function experiments on the PER3 gene and found that silencing it increased the ability of breast cancer cells to proliferate, invade, and metastasize in vitro. Conversely, overexpression of PER3 had an inhibitory effect on these malignant phenotypes of breast cancer cells. Wang et al. [29] demonstrated that the expression level of PER3 was lower in both colon and rectal tumor tissues compared to normal tissues. The GEO database screening of glioblastoma cases also revealed a low expression of the PER3 gene.

The functional annotation of the differential genes showed that the differential genes related to PER3 were enriched in GBM tissues for biological processes such as humoral immune response, phagocytosis, and complement activation, cellular components such as immunoglobulin complexes, blood microparticles, and outer plasma membrane, as well as for molecular functions such as antigen binding, immunoglobulin receptor binding, receptor ligand activity, and signaling receptor activation. The GSEA results showed that genes related to PER3 were enriched in sets associated with neurological function and diseases. These findings suggest that decreased PER3 expression may disrupt normal neurological function and contribute to glioblastoma development by affecting the cycle, migration, and invasion of glioblastoma cells.

Our study demonstrated a potential relationship between aberrant expression of PER3 and tumor immune cell infiltration. The level of PER3 expression was negatively correlated with neutrophils, macrophages, Th2 cells, and NK CD56dim cells. Neutrophils play an important role in suppressing tumors by activating immune responses against tumor cells and directly lysing them [30]. Macrophages use phagocytosis as a major mechanism of innate immunity. This process enables them to engulf and kill intracellular tumor cells, as well as their own senescent and abnormal cells [31]. Macrophages are antigen-presenting cells that play a crucial role in the body’s immune defense, self-stabilization, and immune surveillance by initiating the immune response. Th2 cells, a type of CD4+ T cells, contribute to humoral immunity and selectively activate macrophages, mediate the regression of the inflammatory phase, and initiate tissue repair. NK cells can inhibit tumor growth by interacting with dendritic cells, macrophages, and T cells to develop an immune response. CD56dim NK cells, which are cytotoxic, account for more than 90% of all NK cells. Our data show a positive correlation between PER3 expression and central memory T cells, γδ T cells, effector memory T cells, and follicular helper T cells. In 2005, Klebanoff CA et al. [32] demonstrated the super anti-tumor ability of Tcm cells, which have the ability of self-renewal and replication and long survival in vivo. Tem cells, which are mainly located in peripheral tissues, can rapidly produce effector cytokines once re-stimulated by antigen and are considered activated Tcm cells. Tem are activated Tcm cells, mainly located in peripheral tissues, which can rapidly produce effector cytokines once they are re-stimulated by antigen. γδ T cells can directly serve anti-tumor purposes and are also capable of targeting tumor cells through antibody-dependent cytotoxicity (ADCC). T follicular helper cells regulate tumor growth and progression through the chemokine receptor CXCR5 [33,34]. Taken together, our findings indicate that low expression of the PER3 gene is a significant factor in the immune evasion mechanism of GBM cells.

Abnormal expression and function of immune checkpoint molecules can contribute to the development of various diseases. For instance, overexpression or overfunction of immune checkpoint molecules can suppress immune function, weaken the body’s immunity, and increase susceptibility to tumors and other diseases. Key proteins associated with immune escape from tumors include HLA-A, CD276, and TNFRSF4 [35]. CD276 is associated with diseases such as immunodeficiency and neuroblastoma. It may regulate T-cell-mediated immune responses and play a protective role in tumor cells by inhibiting natural killer-mediated cell lysis. Additionally, it can serve as a marker for the detection of neuroblastoma cells [36]. Researchers have extensively studied the pharmacological efficacy of TNFRSF4 inhibitors using a humanized mouse model. Antibodies against human OX40 have shown significant anti-tumor effects (*p* < 0.001). Furthermore, oncogenes are linked to cell proliferation and tumorigenesis. Malignant tumors exhibit high expression of TP53, and TP53 mutations are associated with poor prognosis in several human cancers [37,38]. CASP3 is a crucial indicator for detecting apoptosis, and it is associated with the development of cancers and neurological disorders [39,40]. The study showed that CPEB3 plays a crucial role in memory storage, and it affects neuronal function when its expression is inhibited [41], suggesting that the CPEB3 protein could be a promising target for treating neurodegenerative diseases. Recent studies have considered CPEB3 and CASP3 as candidate oncogenes [42].

DNA methylation is a prevalent epigenetic mechanism that plays a crucial role in tumorigenesis. Alterations in gene methylation status are linked to the development, growth, and progression of cancer. This paper examines the correlation between the methylation level of the PER3 gene and the prognosis of GBM patients. The study found that poorer overall survival was significantly associated with hypomethylation at five CpG islands, including cg23927002, cg17465881, and cg06487986, with these three loci exhibiting the lowest degree of methylation. Furthermore, the study showed that the probability of PER3 gene mutations occurring in GBM patients is less than 3%. Additionally, the study found no association between PER3 gene mutation and OS or DSS in GBM patients. Our study found significant differences in the expression level of PER3 in GBM tissues based on tumor histopathological staging, WHO grade, IDH type, tumor efficacy assessment, OS events, DSS events, and PFI events. Lower PER3 expression was correlated with poorer prognosis and later clinical staging in GBM patients.

The clinical diagnosis of glioblastoma primarily depends on imaging measures, such as CT and MRI. In early diagnosis, diagnostic ROC curves are often used to determine the diagnostic value of a particular factor for the disease and to assess the predictive accuracy, which reflects the relationship between sensitivity and specificity. This study analyzed the diagnostic value of PER3 in GBM by selecting two molecular pathology indicators of GBM from the Glioma Diagnostic and Treatment Guidelines and analyzing the ROC curves together with PER3 as a diagnostic factor. BRAF has been reported to occur in various gliomas [43], and EGFR is one of the diagnostic indicators of grade 4 GBM of the WHO [44]. Both are potential targets for targeted therapy of GBM. Both PER3 and EGFR are potential targets for glioblastoma-targeted therapy. The area under the curve (AUC) is usually taken between 0.5 and 1, with a value closer to 1 indicating greater diagnostic predictiveness. Figure 9A shows that the AUC values of PER3, BEAF, and EGFR are all above 0.9. Additionally, the AUC values of PER3 for predicting the survival rate of GBM patients at both 5 and 7 years are greater than 0.7. These data further suggest that PER3 is a potential diagnostic and prognostic biomarker for GBM [45].

Furthermore, the results of DEHP exposure experiments on zebrafish showed that long-term exposure to DEHP can lead to the adverse effects of downregulation of PER3 gene expression and circadian dysregulation in zebrafish brain tissues, suggesting that DEHP is potentially carcinogenic to organisms. Previous studies showed that the concentration of DEHP in some sections of the Yellow River was as high as 109 μg/L [23], and the highest detected concentration of DEHP in the middle and lower reaches of the Songhua River was 1752 μg/L, with an average detected concentration of 370 μg/L [24]. To summarize, the concentration of DEHP detected in the water environment in some regions of China far exceeded the limit of the standard for surface water in China (8 μg/L) [46]. Therefore, exposure of aquatic organisms in rivers to these concentrations of DEHP may cause some negative effects. The results of the study provide some theoretical basis for future management of DEHP in the water environment.

## 5. Conclusions

The diagnostic and prognostic value of the circadian factor PER3 in GBM was demonstrated in this study. The PER3 gene is expressed at low levels in tumor tissues of GBM patients and regulates GBM progression by modulating the expression of genes involved in the cell cycle and immune response. Additionally, the PER3 gene is associated with the tumor infiltration status of multiple immune cell types and may play a role in the response of GBM patients to immunotherapy. Taken together, these findings suggest that PER3 may serve as a potential therapeutic target and useful diagnostic and prognostic marker for GBM. Additionally, these findings provide further evidence for the carcinogenicity of DEHP. However, further in vivo and in vitro experiments are necessary to validate the results of this study.

## Figures and Tables

**Table 1:** Effects of methylation levels in the CpG sites of the PER3 gene on the prognosis of GBM patients.

CpG Island	HR	*p*-Value
Body-S_Shore-cg00019458	0.744	0.2004
3′UTR-Open_Sea-cg00811891	1.297	0.2570
Body-Island-cg04725166	0.701	0.1295
Body-N_Shore-cg04837231	0.683	0.1097
**TSS1500-Island-cg06487986**	**0.476**	**0.0011**
Body-S_Shore-cg08134286	1.160	0.5408
**5**′**UTR;1stExon-Island-cg08764927**	**0.506**	**0.0013**
Body-Island-cg08926642	0.708	0.1354
Body-S_Shore-cg09168692	0.726	0.1773
Body-S_Shelf-cg09327610	1.200	0.4652
TSS1500-Island-cg11753033	0.795	0.3440
TSS1500-Island-cg11843502	0.811	0.3937
**Body-Open_Sea-cg11978441**	**0.569**	**0.0156**
**TSS200-Island-cg12258811**	**0.524**	**0.0032**
**5**′**UTR;1stExon-Island-cg14204433**	**0.544**	**0.0174**
Body-Island-cg17328665	0.682	0.0949
**TSS1500-Island-cg17465881**	**0.536**	**0.0260**
**Body-Island-cg17724687**	**1.660**	**0.0293**
Body-Open_Sea-cg22507406	0.897	0.6443
**TSS1500-Island-cg23927002**	**0.602**	**0.0223**
Body-S_Shore-cg24454741	0.712	0.1076
Body-Island-cg25514503	1.321	0.2389

Bolded CpG islands are those with *p*-values less than 0.05.

**Table 2:** Clinicopathological characteristics of GBM patients with high- and low-PER3 expression levels.

Characteristics	Low Expression of PER3	High Expression of PER3	*p*-Value
Total number of patients	349	350	
Race, *n* (%)			0.8315
Asian	6 (0.9%)	7 (1%)	
Black or African American	18 (2.6%)	15 (2.2%)	
White	318 (46.4%)	322 (46.9%)	
Histological type, *n* (%)			<0.001
Astrocytoma	66 (9.4%)	130 (18.6%)	
Glioblastoma	153 (21.9%)	15 (2.1%)	
Oligodendroglioma	85 (12.2%)	115 (16.5%)	
Oligoastrocytoma	45 (6.4%)	90 (12.9%)	
WHO grade, *n* (%)			<0.001
G2	62 (9.7%)	162 (25.4%)	
G3	109 (17.1%)	136 (21.4%)	
G4	153 (24%)	15 (2.4%)	
IDH status, *n* (%)			<0.001
WT	185 (26.9%)	61 (8.9%)	
Mut	157 (22.8%)	286 (41.5%)	
Primary therapy outcome, *n* (%)			0.0052
PD	53 (11.4%)	59 (12.7%)	
SD	62 (13.3%)	86 (18.5%)	
PR	17 (3.7%)	48 (10.3%)	
CR	42 (9%)	98 (21.1%)	
OS event, *n* (%)			<0.001
Alive	152 (21.7%)	275 (39.3%)	
Dead	197 (28.2%)	75 (10.7%)	
DSS event, *n* (%)			<0.001
No	156 (23%)	278 (41%)	
Yes	176 (26%)	68 (10%)	
PFI event, *n* (%)			<0.001
No	129 (18.5%)	224 (32%)	
Yes	220 (31.5%)	126 (18%)	

**Table 3:** Logistic regression analysis of the relationship between clinicopathological characteristics and the PER3 expression levels in GBM patients.

Characteristics	Total (N)	OR (95% CI)	*p* Value
WHO grade (G4 and G3 vs. G2)	637	0.221 (0.155–0.314)	<0.001
IDH status (Mut vs. WT)	689	5.525 (3.898–7.830)	<0.001
Primary therapy outcome (PD and SD vs. CR and PR)	465	0.510 (0.345–0.752)	<0.001
Gender (Male vs. Female)	699	0.915 (0.678–1.235)	0.562
Race (Black or African American and White vs. Asian)	686	0.860 (0.286–2.585)	0.788
Age (>60 vs. ≤60)	699	0.271 (0.180–0.408)	<0.001

## Data Availability

The raw data supporting the conclusions of this article will be made available by the authors upon request.

## Supplementary Materials

The following supporting information can be downloaded at: https://www.mdpi.com/article/10.3390/toxics12120835/s1, Table S1: Functional annotations of differentially expressed genes in GBM patients with different PER3 levels; Table S2: Cox regression analysis of the clinical outcomes in GBM patients based on various clinicopathological characteristics including PER3 levels.
