# Supplementary material for: DEHP-Induced Glioblastoma in Zebrafish Is Associated with Circadian Dysregulation of PER3
Source: Toxics. 2024 Nov 21;12(12):835. doi: 10.3390/toxics12120835 (PMC11679192; doi:10.3390/toxics12120835)
Supplement: Supplementary file 1 [file toxics-12-00835-s001.zip › toxics-3296258-supplementary.pdf]

Supplementary Table S1. Functional annotations of differentially expressed genes in GBM patients with different PER3 levels.

ONTOLOGY	ID	Description	GeneRatio	BgRatio	pvalue	p.adjust	qvalue
BP	GO:0006910	phagocytosis, recognition	56/654	102/18800	1.14e-54	4.87e-51	4.36e-51
BP	GO:0006959	humoral immune response	87/654	317/18800	1.36e-53	2.91e-50	2.61e-50
BP	GO:0006958	complement activation, classical pathway	56/654	108/18800	8.83e-53	1.26e-49	1.13e-49
BP	GO:0002455	humoral immune response mediated by circulating immunoglobulin	57/654	121/18800	1.31e-50	1.40e-47	1.26e-47
CC	GO:0019814	immunoglobulin complex	105/673	167/19594	3.34e-112	1.40e-109	1.35e-109
CC	GO:0042571	immunoglobulin complex, circulating	53/673	77/19594	8.47e-60	1.77e-57	1.71e-57
CC	GO:0072562	blood microparticle	45/673	147/19594	1.75e-30	2.44e-28	2.36e-28
CC	GO:0009897	external side of plasma membrane	71/673	455/19594	3.40e-27	3.55e-25	3.43e-25
MF	GO:0003823	antigen binding	81/627	174/18410	4.09e-72	2.48e-69	2.19e-69
MF	GO:0034987	immunoglobulin receptor binding	52/627	80/18410	7.01e-57	2.13e-54	1.88e-54
MF	GO:0048018	receptor ligand activity	59/627	489/18410	2.15e-17	4.37e-15	3.85e-15
MF	GO:0030546	signaling receptor activator activity	59/627	496/18410	4.22e-17	6.41e-15	5.65e-15

Supplementary Table S2. Cox regression analysis of the clinical outcomes in GBM patients based on various clinicopathological characteristics including PER3 levels.

Characteristics	HR for overall survival (95% CI)		HR for disease-specific survival (95% CI)		HR for progression-free interval (95% CI)	
	Univariate	Multivariate	Univariate	Multivariate	Univariate	Multivariate
WHO grade (G4&G3 vs. G2)	5.593***	1.97**	5.765***	1.863*	2.725***	1.185
IDH status (Mut vs. WT)	0.116***	0.359***	0.110***	0.361***	0.150***	0.304***
1p/19q codeletion (Codel vs. Non-codel)	0.225***	0.601	0.200***	0.541*	0.296***	0.599*
Primary therapy outcome (PD&SD vs. CR&PR)	4.868***	3.531***	5.486***	4.362***	2.954***	2.671***

Race (Black or African American&White vs. Asian)	1.188		1.641		1.355	
Age (> 60 vs. <= 60)	4.696***	3.629***	4.528***	3.356***	2.892***	1.818**
PER3(High vs. Low)	0.293***	0.853***	0.288***	0.812	0.412***	0.751
